# Supplementary material for: Fairer AI in ophthalmology via implicit fairness learning for mitigating sexism and ageism
Source: Nat Commun. 2024 Jun 4;15:4750. doi: 10.1038/s41467-024-48972-0 (PMC11150422; doi:10.1038/s41467-024-48972-0)
Supplement: Supplementary file 1 — Supplementary Information file [file 41467_2024_48972_MOESM1_ESM.pdf]

Supplementary Information: Fairer AI in Ophthalmology via Implicit Fairness Learning for Mitigating Sexism and Ageism

Weimin Tan[1,3], Qiaoling Wei[2,3], Zhen Xing[1], Hao Fu[1], Hongyu Kong[2], Yi Lu[2*], Bo Yan[1*], Chen Zhao[2*]

[1]School of Computer Science, Shanghai Key Laboratory of Intelligent Information Processing, Fudan University, Shanghai, China.
[2]Department of Ophthalmology, Eye and ENT Hospital, Shanghai Medical College, Fudan University, Shanghai, China.
[3]These authors contributed equally.
*Email: dr_zhaochen@fudan.edu.cn; byan@fudan.edu.cn; luyieent@126.com

Table of Contents

Supplementary Figure 1 shows the detailed architecture of FairerOPTH.
Supplementary Figure 2 shows the visualization of class activation maps [1] of FairerOPTH.
Supplementary Table 1 shows a detailed comparison of our datasets with public fundus image datasets.
Supplementary Table 2 shows the false positive rate (FPR) and false negative rate (FNR) of FairerOPTH/Baseline for diseases with imbalanced data.
Supplementary Table 3 shows the evaluation of the ability of FairerOPTH to adapt to text modality.
Supplementary Table 4 shows the diagnostic performance on the public IDRiD dataset without fundus feature annotations.
Supplementary Table 5 shows the results of the ablation study for FairerOPTH.
Supplementary Table 6 shows the exploration results of the contributing potential factors in the causal relationship between fundus features and diseases to mitigate model unfairness.
Supplementary Table 7 shows the classification performance for the retinal pathological features on the OculoScope and MixNAF datasets.
Supplementary Table 8 shows the demographic distribution of ultra-widefield fundus photos in OculoScope.
Supplementary Table 9 shows the division of training, validation and test datasets.

Deployment generalizability of FairerOPTH

The trained FairerOPTH model, like other common fundus image based diagnostic models, can be directly deployed on fundus datasets without fundus feature annotations. As shown in Supplementary Fig. 1, FairerOPTH contains the upper and bottom two branches, where the upper branch is used to extract pathological features and help the bottom branch of disease classification. During the testing phase, similar to other fundus-based disease classification models, the trained FairerOPTH model only needs to input fundus images and can automatically extract pathological features and identify diseases. Note that users would need to provide pathological annotations if they are to retrain or fine-tune the FairerOPTH model for their particular tasks.

FPR and FNR rates of each disease within each group

The FPR and FNR for diseases with imbalanced data are demonstrated in Supplementary Table 2. Cataract, PM and RP have higher incidence rates as age increases, while Coats and FEVR have higher incidence rates at younger ages. There is an obvious data imbalance for these diseases.

The adaptability of FairerOPTH to text modality

Other types of data such as commonly used text can be easily adapted to FairerOPTH. To validate it, additional experiments using text as input to the pathology classification branch were conducted on the OculoScope dataset and denoted as "FairerOPTH (text)". We used the trained BERT (a famous language model) to extract feature from the inputted text. The experimental results are shown in Supplementary Table 3. The "FairerOPTH (text)" and "FairerOPTH (image)" achieve better performance than the baseline. In addition, "FairerOPTH (text)" performs better in screening accuracy and fairness than "FairerOPTH (image)" most of the time. The results demonstrate that FairerOPTH can adapt to the text modality and even achieve better performance.

Classification performance for the retinal pathological features

The classification performance for the retinal pathological features on the OculoScope and MixNAF datasets is demonstrated in Supplementary Table 7. Our FairerOPTH achieves

0.951 and 0.948 AUC (Area Under the Curve) on the OculoScope and MixNAF datasets, respectively.

Theoretic analysis

To theoretically prove that the additional introduction of fundus features related to fundus diseases can improve the performance of the model, we explain it from the perspective of information theory. Shannon entropy [4] is employed to quantify the level of randomness associated with a discrete random variable Y, which has possible outcomes $(y_1, y_2, \cdots, y_n)$. The disease identification model processes a single fundus image and produces predicted probabilities for various ophthalmic diseases, denoted as $(P_1, P_2, \cdots, P_n)$, corresponding to the 38 categories represented in the OculoScope dataset and 16 categories represented in the MixNAF dataset. The entropy $H(Y)$ can be formulated as:

$$\mathrm{H}(Y) = -\sum_{j=1}^{n} P\left(y_j\right) \log P\left(y_j\right) \tag{1}$$

where n denotes the total number of categories. Eq. (1) represents the calculation of the overall confidence of a model across all classes. Considering a disease identification model, where the input features are denoted by X (fundus images), and the output class labels are represented by Y, we introduce additional information C (fundus features), which is closely related to the fundus disease identification task.

In information theory, the mutual information measures the mutual dependence between two random variables. For X and Y, the mutual information $I(X;Y)$ is defined as:

$$I(X;Y) = H(Y) - H(Y|X) \tag{2}$$

where $H(Y)$ is the entropy of Y, and $H(Y|X)$ is the conditional entropy of Y given X.

Similarly, the mutual information between X, Y, and C is given by:

$$I(Y;X,C) = H(Y) - H(Y|X,C) \tag{3}$$

where $H(Y|X,C)$ is the conditional entropy of Y given both X and C.

Then, we aim to prove that when adding the additional information C, which is closely related to the classification task, the conditional entropy $H(Y|X)$ is greater than the conditional entropy $H(Y|X,C)$, $i.e.$, $H(Y|X) > H(Y|X,C)$. Specifically, we start by considering the mutual information $I(Y;X,C)$. The definition of conditional entropy is used as follows:

$$H(Y|X,C) = -\sum P(y|x,c)\log(P(y|x,c)) \tag{4}$$

where $P(y|x,c)$ represents the probability of Y taking the value y given both X and C.

Next, the definition of conditional entropy is used:

$$H(Y|X) = -\sum P(y|x)\log(P(y|x)) \tag{5}$$

where $P(y|x)$ represents the probability of Y taking the value y given X.

Finally, we compare the difference between $I(Y;X,C)$ and $I(Y;X)$:

$$\begin{aligned} I(Y;X,C) - I(Y;X) &\\ &= [H(Y) - H(Y|X,C)] - [H(Y) - H(Y|X)] \\ &= H(Y|X) - H(Y|X,C) \end{aligned} \tag{6}$$

Notably, the term $H(Y)$ is cancelled out in this comparison.

Based on the definition of conditional entropy, we can state that for any y, x, and c, we have $P(y|x,c) \geq P(y|x)$, which implies $H(Y|X) \geq H(Y|X,C)$. Therefore, $H(Y|X) - H(Y|X,C) \geq 0$. Consequently, we conclude that $I(Y;X,C) - I(Y;X) \geq 0$, and thus $I(Y;X,C) \geq I(Y;X)$.

Hence, we have successfully proven, from an information-theory perspective, that when incorporating additional information C, which is closely related to the disease identification task, the conditional entropy $H(Y|X)$ is greater than the conditional entropy $H(Y|X,C)$, $i.e.$, $H(Y|X) \geq H(Y|X,C)$. This indicates that given X, the introduction of C reduces the uncertainty in predicting Y, enhancing the model's predictive capacity and ultimately leading to improved performance of the disease identification model (see below).

Generalization analysis

The reason why reductions in uncertainty can lead to improvements in model performance drives us to delve into the fundamental concept of the generalization bound of FairerOPTH, particularly in how it compares to conventional fundus disease identification models. The generalization bound characterizes the model's ability to transfer knowledge from the training data to unseen testing data. In this context, $V(h)$ represents the generalization error ($i.e.$, testing loss), and $\hat{V}_S(h)$ signifies the empirical error ($i.e.$, training loss) of n independent and identical distribution samples. While the Vapnik-Chervonenkis (VC) dimension [5] traditionally measures the model's capacity to perform binary classification algorithms, here we need to adopt the pseudo-dimension D_{psd} [5] for disease identification tasks, as it aptly describes the generalization bound of infinite hypothesis sets [6], offering an appropriate measure of the model's capacity.

Consider a family of functions H associated with the network that incorporates the fundus features, and let L represent the loss function for disease identification. We have a family of bounded loss functions $\mathcal{F} = \{(x,y) \mapsto \mathcal{L}(h(x),y)\}$ with values in the range [0, K], which are related to H [6]. Assuming that the pseudo-dimension of $\mathcal{F}$ is $d_{\mathrm{FairerOPTH}}$, for any $\sigma > 0$, with a probability of at least $1 - \sigma$, the following inequality holds for all $h \in H$:

$$\begin{aligned} \mathcal{V}(h) \leq \hat{\mathcal{V}}_S(h) + K\sqrt{\frac{2d_{FairerOPTH}\log\frac{R\cdot n}{d_{FairerOPTH}}}{n}} \\ + K\sqrt{\frac{\log\frac{1}{\sigma}}{2n}} \end{aligned} \tag{7}$$

where R denotes a natural constant. This theorem demonstrates that a larger sample size n and a lower pseudo-dimension (D_{psd}) ensures better generalization. As above discussion, the input entropy of the FairerOPTH is lower than that of the conventional identification model, resulting in a reduced number of possible states or choices within the function set H. Consequently, the smaller function sets also contribute to a lower pseudo-dimension [5]. We denote the pseudo-dimension of conventional identification model as $d_{\mathrm{convention}}$; it follows that $d_{\mathrm{FairerOPTH}} \leq d_{\mathrm{convention}}$. As a result, FairerOPTH achieves a lower generalization bound, indicat-

ing its stronger generalizability, which is further corroborated by the experimental results.

References

[1] B. Zhou et al. Learning deep features for discriminative localization. In *Proceedings of the IEEE conference on computer vision and pattern recognition* (2016), pp. 2921–2929.

[2] A. Dosovitskiy et al. An image is worth 16x16 words: Transformers for image recognition at scale. In *International Conference on Learning Representations* (2021).

[3] T. Ridnik et al. Asymmetric loss for multi-label classification. In *Proceedings of the IEEE/CVF International Conference on Computer Vision* (2021), pp. 82–91.

[4] J. Lin. Divergence measures based on the shannon entropy. *IEEE Transactions on Information Theory* **37** no. 1, pp. 145–151 (1991).

[5] M. Mohri, A. Rostamizadeh and A. Talwalkar. *Foundations of Machine Learning*. 2 ed. (MIT Press, 2018).

[6] Z. Li et al. Information-growth attention network for image super-resolution. In *Proceedings of the 29th ACM International Conference on Multimedia* (2021), p. 544–552.

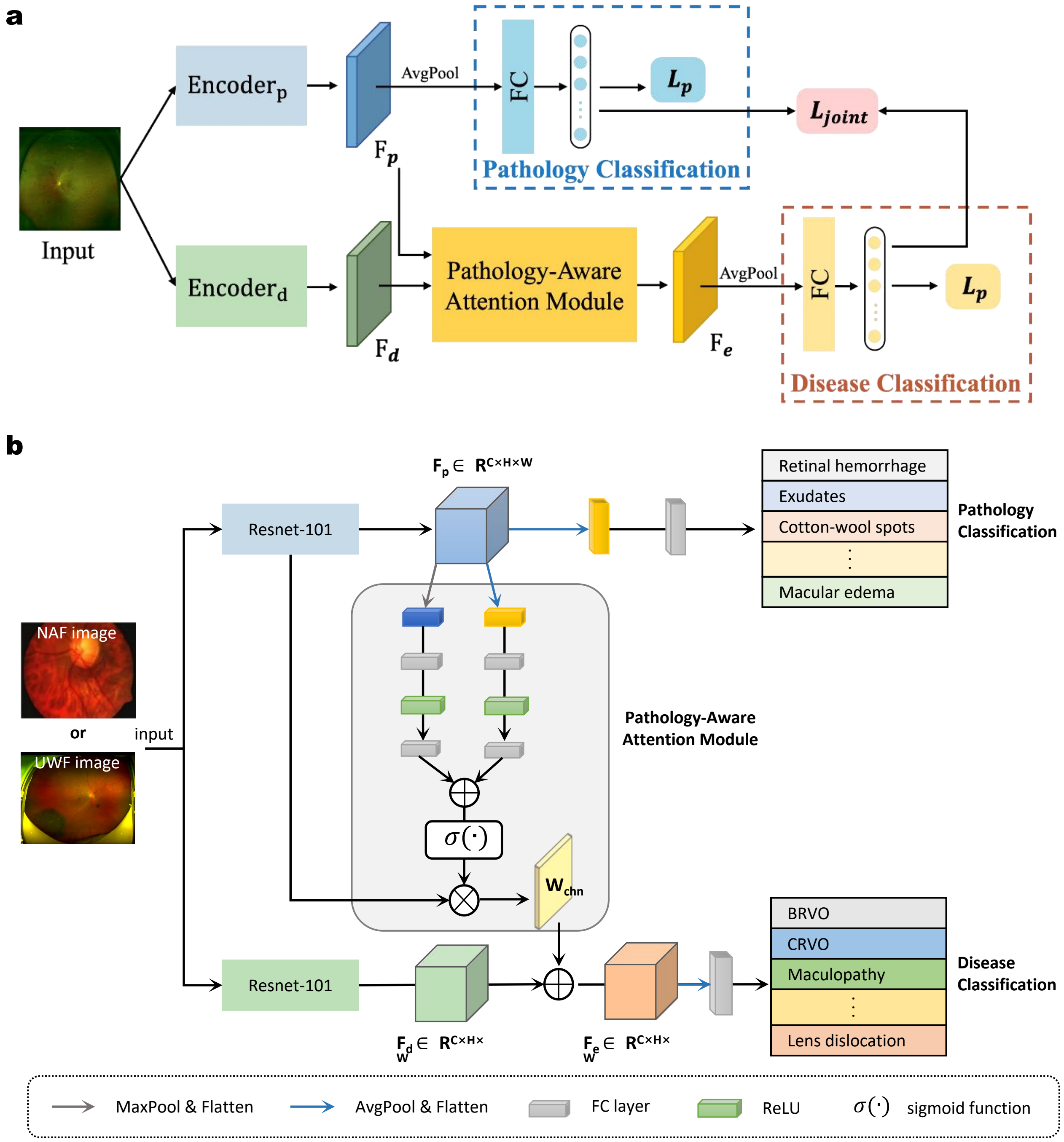

Supplementary Figure 1 | The overall architecture of the proposed FairerOPTH model. **a**, FairerOPTH consists of two branches: a pathology classification branch and disease classification branch. The interaction between these two branches is implemented by a pathology-aware attention module that enhances the fundus feature representations to inform the disease classification branch. Four loss terms including L_{patho}, L_{disea}, L_{joint}, and L_{consis} compose the overall loss to optimize the entire network. **b**, Detailed network structure of the FairerOPTH. We use ResNet-101 as the encoder to extract fundus features. The pathology-aware attention module is implemented with two fully connected layers and one sigmoid function. The implementation of the FairerOPTH is very simple but highly effective in mitigating unfairness and improving screening accuracy.

Supplementary Table 1 | Comparison of our dataset with public fundus image datasets. The proposed MixNAF and OculoScope datasets have unique advantages in terms of the labelling of fundus features. They contain 20 and 67 types of fundus features, respectively.

Dataset	Task	Equipment	Retinal imaging	Fundus image size	Number of patients	Number of images	Types of disease labeling	Types of pathology labeling	Link
Kaggle-EyePACS	Diabetic retinopathy grading	nonmydriatic digital retinal camera	Narrow-field	(433, 289, 3) to (5184, 3456, 3)	44,315	88,702	Five grades of diabetic Retinopathy	None	https://www.kaggle.com/c/diabetic-retinopathy-detection/data
IDRiD	DR staging, DME staging	Kowa VX-10α digital fundus camera	Narrow-field	(2848, 4288, 3)	-	516	Five grades of diabetic Retinopathy; Three DME grades	None	https://ieee-dataport.org/open-access/indian-diabetic-retinopathy-image-dataset-idrid
OIA-DDR	DR staging	Topcon D7000, Topcon TRC NW48, Nikon D5200 and Canon CR 2 cameras	Narrow-field	(1088,1088,3) to (3456,3456,3)	9,598	13,673	Five grades of diabetic Retinopathy	None	https://dl.acm.org/doi/abs/10.1016/j.ins.2019.06.011
OIA-ODIR	8 disease classifications	Canon, Zeiss and Kowa	Narrow-field	(188,250,3) to (3456,5184,3)	5,000	10,000	Eight categories: Normal, diabetes, glaucoma, cataract, AMD, hypertension, myopia and other diseases	None	https://github.com/nkicsl/OIA-ODIR
Messidor	DR staging, AMD diagnosis	colour video 3CCD camera	Narrow-field	1440 × 960, 2240 × 1488, or 2304 × 1536	-	1,200	Four retinopathy grades; Three DME grades	None	https://www.adcis.net/en/third-party/messidor/
Messidor-2	DR staging	-	Narrow-field	-	874	1,748	Five grades of diabetic Retinopathy	None	https://www.adcis.net/fr/logiciels-tiers/messidor2-fr/
LAG	Glaucoma diagnosis	-	Narrow-field	(500, 500, 3)	-	1,200	Suspicious and negative glaucoma	None	https://github.com/smilell/AG-CNN
iChallenge-AMD	AMD diagnosis	Zeiss Visucam 500 fundus camera, Canon CR-2	Narrow-field	(1444, 1444, 3), (2124, 2056, 3)	-	1,200	AMD and non-AMD	None	https://aistudio.baidu.com/aistudio/datasetdetail/88462
iChallenge-PM	PM diagnosis	-	Narrow-field	-	1,200	1,200	Pathological Myopia and non Pathological Myopia	None	https://aistudio.baidu.com/aistudio/datasetdetail/88464
iChallenge-GON	Glaucoma diagnosis	Zeiss Visucam 500, Canon CR-2	Narrow-field	(2124, 2056, 3), (1634, 1634, 3)	-	1,200	Glaucoma and non glaucoma	None	https://aistudio.baidu.com/aistudio/datasetdetail/88463
RFMiD	46 disease classifications	TOPCON 3D OCT-2000, Kowa VX-10, TOPCON TRC-NW300	Narrow-field	(4288, 2848, 3) (2144, 1424, 3) (2048, 1536, 3)	-	3,200	46 categories	None	https://riadd.grand-challenge.org/download-all-classes/
**Our MixNAF**	**Fairer AI in Ophthalmology**	**-**	**Narrow-field**	**(433, 289,3) to (5184, 3456, 3)**	**-**	**4,540**	**16 categories**	**20 categories**	**Described in the data availability section**
**Our OculoScope**	**Fairer AI in Ophthalmology**	**Optos P200dTx (Optos PLC, Dunfermline, United Kingdom)**	**Ultra-widefield**	**(3900, 3072, 3)**	**8,405**	**16,530**	**38 categories**	**67 categories**	**Described in the data availability section**

Supplementary Table 2 | Demonstration of the false positive rate (FPR) and false negative rate (FNR) of FairerOPTH/Baseline for diseases with imbalanced data. Cataract, PM and RP have higher incidence rates as age increases, while Coats and FEVR have higher incidence rates at younger ages. There is an obvious data imbalance for these diseases.

Disease	Age Group	FPR	FNR
	0~10	none	none
	10~20	0.0/0.007	0.0/0.0
	20~30	0.036/0.197	0.0/0.0
	30~40	0.096/0.163	0.0/0.0
Cataract	40~50	0.11/0.247	0.154/0.0
	50~60	0.095/0.152	0.1/0.1
	60~70	0.094/0.165	0.072/0.105
	70~80	0.071/0.283	0.103/0.115
	80~90	0.0/0.1	0.0/0.0
	0~10	none	none
	10~20	0.0/0.0	0.0/0.0
	20~30	0.027/0.007	0.0/0.0
	30~40	0.033/0.048	0.0/0.0
PM	40~50	0.02/0.017	0.026/0.0
	50~60	0.023/0.034	0.028/0.028
	60~70	0.015/0.019	0.06/0.03
	70~80	0.026/0.036	0.0/0.0
	80~90	0.125/0.125	0.0/0.0
	0~10	0.0/0.0	0.0/0.0
	10~20	0.0/0.0	0.0/0.0
	20~30	0.0/0.0	0.0/0.0
	30~40	0.0/0.0	0.0/0.0
RP	40~50	0.0/0.0	0.0/0.0
	50~60	0.0/0.0	0.0/0.0
	60~70	0.0/0.0	0.0/0.0
	70~80	0.0/0.0	0.0/0.0
	80~90	none	none
	0~10	0.01/0.0	0.0/0.0
	10~20	0.008/0.008	0.0/0.0
	20~30	0.0/0.0	0.0/0.0
	30~40	0.0/0.0	0.0/0.0
Coats	40~50	0.0/0.003	0.0/0.0
	50~60	0.479/0.074	0.0/0.0
	60~70	none	none
	70~80	none	none
	80~90	none	none
	0~10	0.0/0.037	0.0/0.0
	10~20	0.007/0.014	0.0/0.0
	20~30	0.0/0.0	0.0/0.0
	30~40	0.0/0.003	0.0/0.0
FEVR	40~50	none	none
	50~60	none	none
	60~70	none	none
	70~80	none	none
	80~90	none	none

PM, Pathological Myopia; RP, Retinitis Pigmentosa; FEVR, Familial Exudative Vitreoretinopathy.

Supplementary Table 3 | Evaluation of the ability of FairerOPTH to adapt to text modality. "FairerOPTH (text)" denotes the input to the pathology classification branch is the text description of fundus features. Four screening accuracy and fairness metrics are shown.

Method	Screening accuracy metrics				Fairness metrics							
					Age (10 years)				sex			
	mAP(%)	Spec.	Sens.	AUC	A↓ (%)	M↓ (%)	DPM↑ (%)	EOM↑ (%)	A↓ (%)	M↓ (%)	DPM↑ (%)	EOM↑ (%)
Baseline	87.9	0.957	0.951	0.982	44.3	131.8	39.2	91.1	10.3	60.5	71.2	96.1
**FairerOPTH (text)**	**94.6**	**0.981**	**0.968**	**0.991**	**24.5**	125.7	**60.0**	92.5	**4.7**	**48.6**	80.9	97.3
**FairerOPTH (image)**	92.0	0.970	0.956	0.986	31.9	**122.7**	49.6	**93.4**	7.6	66.5	**82.4**	**97.6**

Supplementary Table 4 | Diagnostic performance on the public IDRiD dataset without fundus feature annotations.

Method	Average precision	Average accuracy	AUC
Baseline	0.749	0.587	0.535
ViT-Large [2]	0.772	0.664	0.670
FairerOPTH	0.840	0.694	0.734

Supplementary Table 5 | Ablation study. The ablation study was performed on the OculoScope and MixNAF datasets for both the proposed pathology-aware attention module and feature consistency loss.

Dataset	Method	Backbone	mAP	Spec.	Sens.	AUC
	ASL [3]	ResNet-101	89.7	0.963	0.948	0.982
OculoScope	ASL [3] + Pathology	ResNet-101	90.0	0.964	0.950	0.982
	FairerOPTH	ResNet-101	92.0	0.970	0.956	0.986
	ASL [3]	ResNet-101	82.9	0.944	0.955	0.978
MixNAF	ASL [3] + Pathology	ResNet-101	83.2	0.953	0.947	0.973
	FairerOPTH	ResNet-101	85.1	0.945	0.961	0.980

Supplementary Table 6 | Exploration of the contribution of potential factors in the causal relationship between fundus features and diseases to mitigate model unfairness. The average screening accuracy AP↑ and four fairness metrics (ΔD, PQD, DPM, and EOM) are used to evaluate the capability of FairerOPTH/Baseline to screen different age groups (10 years division) of patients.

Factor	Disease	AP ↑ (%)	ΔD ↓ (%)	PQD ↑ (%)	DPM ↑ (%)	EOM ↑ (%)
Congenital diseases with different manifestations in different periods	Inherited Retinal Diseases (including retinitis pigmentosa, stargardt, and other congenital macular diseases)	96.3/92.9	17.4/17.0	96.1/92.9	36.4/28.7	97.6/88.3
Mixed cause	Optic abnormalities	70.2/67.6	37.2/37.5	91.3/80.2	37.4/17.9	83.3/81.8
Great individual difference	Fibrosis	71.3/60.2	85.9/88.4	88.0/89.6	23.3/19.4	80.0/78.9

Supplementary Table 7 | Classification performance for the retinal pathological features on the OculoScope and MixNAF datasets. Accurately classifying the 67 categories of retinal pathological features in OculoScope and the 20 categories in MixNAF is greatly challenging. Our FairerOPTH achieves decent classification performance.

Retinal pathological features	Sensitivity	Specificity	AUC
OculoScope (ultra-widefield)	0.939	0.911	0.951
MixNAF (narrow-angle)	0.910	0.927	0.948

Supplementary Table 8 | Demographic Distribution of Ultra-Widefield Fundus Photos in the OculoScope dataset.

Parameter	Total or Mean (range)
No. of images	16,530
No. of test images from male(female)*	1,233/1,442
No. of patients	8,405
Sex (n, male/female) of OculoScope	11/41
Age (yrs)# of OculoScope	26.7±6.2 (17~44)

* The sex of patients was initially recorded based on their ID card information and was subsequently confirmed by doctors and operating technicians, taking into consideration the patients' physical and physiological characteristics.

# Patient age was recorded on the day of fundus photography.

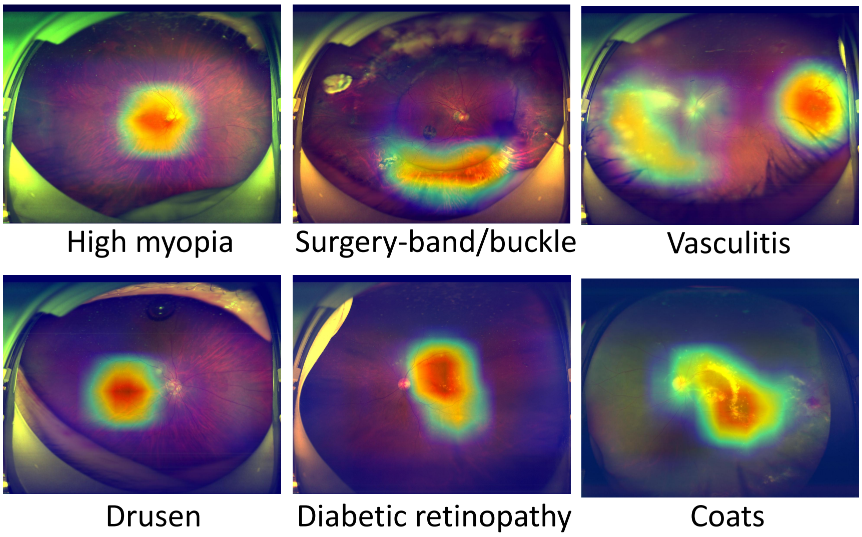

Supplementary Figure 2 | Visualization of class activation maps [1] of FairerOPTH.

Supplementary Table 9 | The division of training and testing datasets.

Dataset	Training set	Testing set
OculoScope	13,855	2,675
MixNAF	3,614	926
