# Supplementary material for: Fairer AI in ophthalmology via implicit fairness learning for mitigating sexism and ageism
Source: Nat Commun. 2024 Jun 4;15:4750. doi: 10.1038/s41467-024-48972-0 (PMC11150422; doi:10.1038/s41467-024-48972-0)
Supplement: Supplementary file 2 — Reporting Summary [file 41467_2024_48972_MOESM2_ESM.pdf]

nature portfolio

Corresponding author(s): Yi Lu, Bo Yan, Chen Zhao

Last updated by author(s): Mar 21, 2024

Reporting Summary

Nature Portfolio wishes to improve the reproducibility of the work that we publish. This form provides structure for consistency and transparency in reporting. For further information on Nature Portfolio policies, see our Editorial Policies and the Editorial Policy Checklist.

Statistics

For all statistical analyses, confirm that the following items are present in the figure legend, table legend, main text, or Methods section.

n/a	Confirmed	
☐	☒	The exact sample size (*n*) for each experimental group/condition, given as a discrete number and unit of measurement
☐	☒	A statement on whether measurements were taken from distinct samples or whether the same sample was measured repeatedly
☒	☐	The statistical test(s) used AND whether they are one- or two-sided<br>*Only common tests should be described solely by name; describe more complex techniques in the Methods section.*
☒	☐	A description of all covariates tested
☐	☒	A description of any assumptions or corrections, such as tests of normality and adjustment for multiple comparisons
☒	☐	A full description of the statistical parameters including central tendency (e.g. means) or other basic estimates (e.g. regression coefficient) AND variation (e.g. standard deviation) or associated estimates of uncertainty (e.g. confidence intervals)
☒	☐	For null hypothesis testing, the test statistic (e.g. *F*, *t*, *r*) with confidence intervals, effect sizes, degrees of freedom and *P* value noted<br>*Give P values as exact values whenever suitable.*
☒	☐	For Bayesian analysis, information on the choice of priors and Markov chain Monte Carlo settings
☒	☐	For hierarchical and complex designs, identification of the appropriate level for tests and full reporting of outcomes
☒	☐	Estimates of effect sizes (e.g. Cohen's *d*, Pearson's *r*), indicating how they were calculated

Our web collection on statistics for biologists contains articles on many of the points above.

Software and code

Policy information about availability of computer code

Data collection	The following tools were used: Matlab 2020a, OPTOMAP.
Data analysis	The following tools were used:<br>Python 3.7 for performing enrichment tests<br>PyCharm Professional 2020.02 for plotting figures<br>PyTorch 1.10.1 and Torchvision 0.11.2 for building and training deep learning models<br>Numpy 1.19.2 for numerical calculation and analysis<br>Seaborn 0.11.1 for data visualization<br>The pytorch code of our FairerOPTH, together with trained models, as well as some example images for testing are publicly available at https://github.com/mintanwei/Fairer-AI (https://doi.org/10.5281/zenodo.10892893).

For manuscripts utilizing custom algorithms or software that are central to the research but not yet described in published literature, software must be made available to editors and reviewers. We strongly encourage code deposition in a community repository (e.g. GitHub). See the Nature Portfolio guidelines for submitting code & software for further information.

Data

Policy information about availability of data

All manuscripts must include a data availability statement. This statement should provide the following information, where applicable:

- Accession codes, unique identifiers, or web links for publicly available datasets
- A description of any restrictions on data availability
- For clinical datasets or third party data, please ensure that the statement adheres to our policy

This study utilizes some public datasets, including JSIEC (https://zenodo.org/record/3477553), Kaggle-EyePACS (https://www.kaggle.com/c/diabetic-retinopathy-detection), iChallenge-AMD (https://ai.baidu.com/broad/download), OIA-ODIR (https://odir2019.grand-challenge.org/), and RFMiD (https://riadd.grand-challenge.org/download-all-classes/). The public IDRiD dataset can be accessed through the following link: https://ieee-dataport.org/open-access/indian-diabetic-retinopathy-image-dataset-idrid. Our newly collected OculoScope and reorganized MixNAF datasets can be accessed through the following link: https://drive.google.com/drive/folders/1XUaillgNC0Xx60fdxMn-FfffFMNMU-jg?usp=drive_link for MixNAF dataset and https://zenodo.org/records/10403889 for OculoScope dataset.

Research involving human participants, their data, or biological material

Policy information about studies with human participants or human data. See also policy information about sex, gender (identity/presentation), and sexual orientation and race, ethnicity and racism.

Reporting on sex and gender	Our study has strictly followed the 'Sex and Gender Equity in Research – SAGER – guidelines'. We acknowledge the significance of this topic in ensuring the ethical and comprehensive conduct of research.<br>Data Collection Process. In our study, patient sex (biological attribute) and age were meticulously collected based on physical and physiological characteristics. The sex and age data were extracted from the electronic patient records system at the Ear, Nose, and Throat (EENT) hospital. To provide further clarity on our data collection process:<br>- Initially, sex and age registration within our hospital's record system is completed by our dedicated registration department staff. This registration relies on the sex and birth date information as indicated on the patient's official ID card issued by the People's Republic of China.<br>- Subsequently, before any treatment is administered, our medical practitioners play a pivotal role in not only verifying the patient's name but also cross-referencing and confirming the recorded sex and age. This rigorous verification step is of paramount importance, especially when disparities are observed between the registered sex and the patient's biological sex. In cases where such disparities emerge (e.g., when patients utilize a relative or friend's ID for outpatient registration), the recorded sex is adjusted after a comprehensive confirmation process with the patient. This ensures the accurate representation of the patient's biological sex in our records.<br>- Furthermore, during the fundus photo examination, our highly trained operating technicians perform another verification step by reconfirming the patient's name, age, and sex. This additional layer of scrutiny guarantees that each fundus photo is correctly attributed to the corresponding patient, thereby eliminating any potential sources of error in sex identification.
Reporting on race, ethnicity, or other socially relevant groupings	None
Population characteristics	In this study, we collected the largest and most diverse fundus image dataset with data from over 8,405 patients representing a wide age range (0 to 90 years). The number of wide-angle fundus test images corresponding to male and female (biological attribute) are 1233 and 1442, respectively.
Recruitment	In this study, we retrospectively collected fundus photos of patients who have visited our hospital based on their diagnosed fundus diseases. However, the dataset has an imbalance in the number of images across diseases, with some being overrepresented and others having limited data.This imbalance may introduce self-selection bias, potentially affecting the model's performance. Diseases with more images may be better predicted, while rare diseases with fewer data points may have lower accuracy. Despite efforts to address this bias, it's important to interpret the results with this limitation in mind, particularly in real-world applications where rare diseases are concerned.
Ethics oversight	Ethical approval for the study was obtained from the Ethics Committee of Shanghai Eye and ENT Hospital（no.2023427) and all procedures were in accordance with the Declaration of Helsinki. Rigorous measures were implemented to anonymize patient information, ensuring the protection of patient confidentiality.

Note that full information on the approval of the study protocol must also be provided in the manuscript.

Field-specific reporting

Please select the one below that is the best fit for your research. If you are not sure, read the appropriate sections before making your selection.

[x] Life sciences [ ] Behavioural & social sciences [ ] Ecological, evolutionary & environmental sciences

For a reference copy of the document with all sections, see nature.com/documents/nr-reporting-summary-flat.pdf

Life sciences study design

All studies must disclose on these points even when the disclosure is negative.

Sample size	The collected dataset contains 16,530 UWF images and 4,540 NAF images, respectively. This is the largest and most diverse fundus image dataset with data from over 8,405 patients representing a wide age range (0 to 90 years). The number of wide-angle fundus test images corresponding to male and female (biological attribute) are 1233 and 1442, respectively. Therefore, the collected dataset is sufficient to evaluate the performance of fundus diagnostic models based on the differences in multiple diagnostic accuracies and fairness evaluation metrics shown by the experimental results.
Data exclusions	Those fundus images without clear diagnostic result and fundus features will be excluded.
Replication	The implementation of the proposed FairerOPTH method is very simple but highly effective in mitigating unfairness and improving screening accuracy. The codes of FairerOPTH are freely available at https://github.com/mintanwei/Fairer-AI.
Randomization	The fundus images are randomly selected from the hospital database. The training and test samples are randomly divided according to the 8:2 ratio.
Blinding	Following existing AI research, this study involves a random division of the dataset. The diagnostic models were blindly tested using the data that were not included in the training process.

Reporting for specific materials, systems and methods

We require information from authors about some types of materials, experimental systems and methods used in many studies. Here, indicate whether each material, system or method listed is relevant to your study. If you are not sure if a list item applies to your research, read the appropriate section before selecting a response.

Materials & experimental systems

n/a	Involved in the study
☒	☐ Antibodies
☒	☐ Eukaryotic cell lines
☒	☐ Palaeontology and archaeology
☒	☐ Animals and other organisms
☒	☐ Clinical data
☒	☐ Dual use research of concern
☒	☐ Plants

Methods

n/a	Involved in the study
☒	☐ ChIP-seq
☒	☐ Flow cytometry
☒	☐ MRI-based neuroimaging

Plants

Seed stocks	None
Novel plant genotypes	None
Authentication	None
