# Supplementary material for: Fairer AI in ophthalmology via implicit fairness learning for mitigating sexism and ageism
Source: Nat Commun. 2024 Jun 4;15:4750. doi: 10.1038/s41467-024-48972-0 (PMC11150422; doi:10.1038/s41467-024-48972-0)
Supplement: Supplementary file 4 — Description of Additional Supplementary Files [file 41467_2024_48972_MOESM4_ESM.pdf]

Supplementary Data 1. List the abbreviated name and number of fundus images for retinal diseases involved.

A. Complete list of abbreviations for retinal diseases involved.

B. Demonstration of the number of images per disease, categorized based on the sex attribute within each of the disease categories.

C. Demonstration of the number of images per disease, categorized based on the age attribute within each of the disease categories.
